# Food Sources of Energy and Nutrients among Adults in the US: NHANES 2003–2006

**DOI:** 10.3390/nu4122097

**Published:** 2012-12-19

**Authors:** Carol E. O’Neil, Debra R. Keast, Victor L. Fulgoni, Theresa A. Nicklas

**Affiliations:** 1 School of Human Ecology, Louisiana State University Agricultural Center, Baton Rouge, LA 70803, USA; 2 Food & Nutrition Database Research, Inc., Shadywood Lane, Okemos, MI 48864, USA; E-Mail: keastdeb@comcast.net; 3 Nutrition Impact, LLC, Battle Creek, MI 49014, USA; E-Mail: vic3rd@aol.com; 4 USDA/ARS Children’s Nutrition Research Center, Department of Pediatrics, Baylor College of Medicine, Houston, TX 77030, USA; E-Mail: tnicklas@bcm.tmc.edu

**Keywords:** NHANES, energy intake, nutrients, nutrient intakes adults, food sources

## Abstract

Identification of current food sources of energy and nutrients among US adults is needed to help with public health efforts to implement feasible and appropriate dietary recommendations. To determine the food sources of energy and 26 nutrients consumed by US adults the 2003–2006 National Health and Nutrition Examination Survey (NHANES) 24-h recall (Day 1) dietary intake data from a nationally representative sample of adults 19+ years of age (y) (*n* = 9490) were analyzed. An updated USDA Dietary Source Nutrient Database was developed for NHANES 2003–2006 using current food composition databases. Food grouping included ingredients from disaggregated mixtures. Mean energy and nutrient intakes from food sources were sample-weighted. Percentages of total dietary intake contributed from food sources were ranked. The highest ranked sources of energy and nutrients among adults more than 19 years old were: energy—yeast bread/rolls (7.2%) and cake/cookies/quick bread/pastry/pie (7.2%); protein—poultry (14.4%) and beef (14.0%); total fat—other fats and oils (9.8%); saturated fatty acids—cheese (16.5%) and beef (9.1%); carbohydrate—soft drinks/soda (11.4%) and yeast breads/rolls (10.9%); dietary fiber—yeast breads/rolls (10.9%) and fruit (10.2%); calcium—milk (22.5%) and cheese (21.6%); vitamin D—milk (45.1%) and fish/shellfish (14.4%); and potassium—milk (9.6%) and coffee/tea/other non-alcoholic beverages (8.4%). Knowledge of primary food sources of energy and nutrients can help health professionals design effective strategies to reduce excess energy consumed by US adults and increase the nutrient adequacy of their diets.

## Abbreviations

CHD: coronary heart disease
CSFII: Continuing Survey of Food Intake by Individuals
DFE: dietary folate equivalents
DSN: Dietary Source Nutrient
DGA: Dietary Guidelines for Americans
FNDDS: Food and Nutrient Database for Dietary Studies
IOM: Institute of Medicine
MUFA: monounsaturated fatty acids
NHANES: National Health and Nutrition Examination Survey
PUFA: polyunsaturated fatty acids
RTEC: ready-to-eat cereal
SE: standard error
SFA: saturated fatty acids
SR: standard reference
US: United States
USDA: United States Department of Agriculture
WWEIA: What We Eat in America
y: years

## 1. Introduction

According to the most recently available data, the age-adjusted prevalence of overweight and obesity in adults was 68% [1]. Consumption of excess energy without a concomitant increase in physical activity is a major reason why there are extremely high obesity rates in the US [2]. Food availability data from 1970 to 2005 suggests, despite an overabundance of healthful foods, such as whole grains, fruit, vegetables, low-fat dairy, and lean meats, too few of these nutrient-dense foods, and too many energy-dense, nutrient-poor foods/beverages were consumed in the US [3]. This finding has been confirmed using nationally representative data from the National Health and Nutrition Examination Survey (NHANES) [4,5,6,7,8]. 

Consumption of energy-dense, nutrient-poor foods/beverages can increase intake of added sugars [9], fats [9], sodium [10], and alcohol [11] and decrease diet quality [5,12]. Consumption of these foods, in turn, may be associated with increased risk of chronic diseases, including coronary heart disease (CHD) [13], type 2 diabetes [14], and metabolic syndrome [15]. Further, due to low intake of nutrient-dense foods, many adults do not meet the recommendations for certain micronutrients [16]. The 2010 Dietary Guidelines for Americans (DGA) specifically identified dietary fiber, calcium, vitamin D, and potassium as “nutrients of concern” which are underconsumed by the population and present a substantial public health concern [17]. Consumption of dietary fiber has been associated with positive health outcomes, including optimizing gut health [18], reducing risk of cardiovascular mortality [19,20], and modulating blood glucose [21]. Dietary fiber intake has also been linked to lower body weight and protection against weight gain [22,23,24]. Inadequate intake of nutrients, such as calcium and vitamin D, in combination with a sedentary lifestyle can increase the risk of developing osteoporosis [25,26]. Inadequate potassium intake, especially when coupled with high sodium intake, can adversely affect blood pressure [10], and is associated with increased osteoporosis [27], and cardiovascular disease risk [10,28]. The 2010 DGA also identified vitamins A, C, E, and K; choline; magnesium; and folate (in select populations) as shortfall nutrients [17]. In addition to highlighting nutrients of concern and shortfall nutrients, the 2010 DGA also reaffirmed food group intake recommendations, and acknowledged the overconsumption of energy and other dietary components, especially sodium [17]. The recommendation to reduce sodium intake is consistent with a recent Institute of Medicine (IOM) report [29].

Understanding current food selections among the US population is critical for designing strategies to help Americans meet nutrient recommendations within energy needs. The most recent publications that provided a detailed list of the food sources of energy and nutrients among US adults report data from the Continuing Survey of Food Intake by Individuals (CSFII) conducted in 1989 to 1991 [30] and 1994 to 1996 [31]. The purpose of this study was to update previous research by using nationally representative data from the NHANES recently conducted in 2003 to 2006 to examine food sources of energy and 26 nutrients consumed by US adults. 

## 2. Methods

### 2.1. Study Overview

Only food sources of energy and nutrient intake were examined; dietary supplements and medications were excluded from the sources of nutrient intake examined in this study. The 26 nutrients examined in this study included: protein, total fat, saturated fatty acids (SFA), monounsaturated fatty acids (MUFA), polyunsaturated fatty acids (PUFA), cholesterol, carbohydrate, total sugars, dietary fiber, vitamin A, vitamin E, vitamin C, thiamin, riboflavin, niacin, vitamin B6, dietary folate equivalents (DFE), vitamin B12, vitamin D, calcium, phosphorus, magnesium, iron, zinc, sodium, and potassium. The United States Department of Agriculture (USDA) had separated the components of total folate to calculate DFE (which accounts for the greater bioavailability of folic acid compared to food folate). 

### 2.2. Study Population and Dietary Intake

USDA had determined energy and nutrient intake derived from foods consumed using 24-h recall dietary interview data they collected from What We Eat in America (WWEIA) in NHANES 2003–2006 participants with an automated multiple-pass method [32,33]. A detailed description of the dietary interview methods has been described previously [34]. In-person [Day 1] dietary interview data from adults 19 y and older (*n* = 9490) were examined in this study; data judged incomplete or unreliable by the Food Surveys Research Group were excluded from analyses. In additional analyses, the sample was subdivided into two age groups: 19–50 y (*n* = 5429) and 51 y and older (*n* = 4061). Because this study was secondary analysis of public-use data available without personal identifiers, it was exempted from review by the Louisiana State University Agricultural Center Institutional Review Board.

### 2.3. Food Groupings and Composition

The USDA Dietary Source Nutrient (DSN) database [35] was used to define food groups. The DSN database was originally developed for use with the CSFII 1994–1996, thus, for this study, the DSN database had to be updated for application to recent food consumption surveys. Food grouping and disaggregation rules used to update the DSN database were similar to methods reported by others [30,31]. The more than 130 DSN food groups were collapsed into 51 categories (Table 1), an aggregation level consistent with that used by the USDA Food Surveys Research Group when defining food groups [36,37]. Categories of food include survey foods or ingredients of recipes for home- or restaurant-prepared mixtures that were disaggregated including sandwiches; casseroles; ethnic foods; soup; salads; cooked grains, vegetables or meat with added salt, fats, or sauces; and beverage mixtures. None of the baked goods (neither home-baked nor commercially prepared baked goods), and none of the manufactured foods were disaggregated. 

If foods were not disaggregated in the DSN database, the Food and Nutrient Database for Dietary Studies (FNDDS) codes were assigned to DSN food groups (FNDDS versions 2.0 [36] and 3.0 [37] were used in 2003–2004 and 2005–2006 NHANES, respectively). The ingredients of disaggregated survey food recipes (coded using the USDA Nutrient Database for Standard Reference (SR) food codes) were linked to the appropriate food composition databases using the SR-Link file of the FNDDS (versions 2.0 [36] and 3.0 [37] link SR releases 18 [38] and 20 [39], respectively). Recipe calculations were performed to determine proportions of the disaggregated survey foods assigned to the 51 DSN food groups. Ingredients of a recipe for a grain-based mixture such as macaroni and cheese, for example, included macaroni, flour, margarine or butter, milk and cheese which were each classified to respective DSN food groups. Ingredients added in food preparation, such as table salt (salt) added to vegetables or used when broiling meat, were disaggregated to separate the sodium that was added from that which was naturally-occurring in foods. Thus effectively, salt added in food preparation was separated from other ingredients by disaggregating the ingredients of survey food recipes, since the DSN food grouping had defined “salt” as a separate category listed with “Other Foods.”

**Table 1:** Categories of foods for food sources analyses.

GRAIN PRODUCTS	FRUIT	FATS & OILS
Flour, bran, baking ingredients	Fruit	Margarine and butter ^e^
Yeast breads and rolls ^a^	Fruit juice	Salad dressings, mayonnaise
Hot breakfast cereal		Other fats and oils ^e^
Ready-to-eat cereal		
Rice, cooked grains	**DAIRY PRODUCTS**	**DESSERTS & SWEETS**
Pasta	Milk	Cake, cookies, quick bread, pastry, pie ^f^
Biscuits, corn bread, pancakes, tortillas ^a^	Milk drinks	Milk desserts ^g^
Crackers, popcorn, pretzels, chips	Yogurt	Candy, sugars and sugary foods ^h^
Mixtures mostly grain ^b^	Cheese	
**VEGETABLES**	**MEAT, POULTRY, FISH**	**BEVERAGES**
Potatoes(white)	Beef	Fruit drinks and ades
Broccoli, spinach, greens	Lamb, veal, game	Soft drinks, soda
Carrots, sweet potatoes, winter squash	Pork, ham, bacon	Coffee, tea, other nonalcoholic beverages
Tomatoes, tomato/vegetable juice	Organ meats	Alcoholic beverages
Lettuce	Frankfurters, sausages, luncheon meats	**OTHER FOODS**
String beans (green, yellow, wax)		Meal replacements/supplements
Corn, peas, lima beans	Poultry	Soup, broth, bouillon
Olives, pickles	Fish and shellfish	Condiments and sauces
Other vegetables	Mixtures mainly meat, poultry, fish ^c^	Whey and artificial sweeteners
Mixed vegetables, vegetable mixtures	**EGGS, LEGUMES, NUTS & SEEDS**	Salt ^i^
	Eggs ^d^	
	Legumes	
	Nuts, seeds (include butters, pastes)	

^a^ Includes ingredients of recipes for home-made yeast breads/rolls, biscuits, and corn bread; ingredients such as eggs and oils added to boxed biscuit and pancake mixes; and commercially-prepared yeast breads/rolls, biscuits, corn bread, pancakes, waffles, and tortillas; ^b^ Includes manufactured foods that were mixtures mostly grain such as frozen pizza, and canned pasta; ^c^ Includes manufactured foods that were mixtures mainly meat, poultry, fish such as frozen meals; ^d^ Excludes eggs found in yeast bread and rolls; pasta; biscuits, corn bread, pancakes, tortillas; salad dressings, mayonnaise; cakes, cookies, quick bread, pastry, pie; and milk desserts (e.g., ice cream, puddings, and custards); ^e^ Butter, margarine, and oils do not include those used in yeast breads and rolls; ready-to-eat cereals; pasta; biscuits, corn bread, pancakes, tortillas; crackers, popcorn, pretzel, chips; salad dressings, mayonnaise; cakes, cookies, quick bread, pastry, pie; and milk desserts (e.g., ice cream, puddings, and custards); ^f^ Includes ingredients of recipes for home-baked and restaurant-prepared cakes, cookies, quick bread, pastry, pie; ingredients such as eggs and oils added to boxed mixes; and manufactured cakes, cookies, quick bread, pastry, pie; ^g^ Milk desserts include ice-cream, puddings that were home-prepared (by adding milk to boxed mixes), ready-to-serve puddings, and custard; ^h^ Sugars excludes sweeteners added to grain products, canned fruit, desserts, fruit drinks/ades, and soft drinks/soda; ^i^ Includes salt used as an ingredient of home- or restaurant-prepared mixtures (sandwiches; casseroles; ethnic foods; soup; salads; and cooked grains, vegetables or meat with added salt, fats, or sauces); and excludes salt added to manufactured foods, such as baked goods; breakfast cereals; snack foods; frozen pizza; canned vegetables/fruit; canned pasta; canned soup; and condiments.

### 2.4. Statistical Analyses

Analyses were conducted using SUDAAN release 9.0.3 (Research Triangle Institute, Research Triangle Park, NC, USA, 2007) [40]. Appropriate weights were used to adjust for oversampling of certain groups, non-response by some selected sample persons, and to adjust for the complex sample design of NHANES to ensure national representative results [41]. Mean and standard errors (SE) of energy and nutrient intakes from the total diet and from each food group were determined using PROC DESCRIPT of SUDAAN. Mean nutrient intake from each food group was expressed as a percentage of the total dietary intake of that nutrient. Percentages of total nutrient intake contributed from food sources were tabulated by ranked order.

## 3. Results

Food sources of energy and 26 nutrients are shown in Table 2, Table 3, Table 4, Table 5, Table 6, Table 7, Table 8, Table 9, Table 10, Table 11, which appear in this article, and Supplemental Tables S1–S17 which are available online. 

### 3.1. Energy, Macronutrients, Cholesterol and Dietary Fiber

Food sources of energy, protein, total fat, SFA, carbohydrate and dietary fiber are summarized in Table 2, Table 3, Table 4, Table 5, Table 6, Table 7, respectively. Food sources of MUFA, PUFA, cholesterol, and total sugars are summarized in Supplemental Tables S1–S4, respectively. 

More than 20 food groups were principal food sources of energy. The two highest ranked food sources, yeast breads/rolls and cake/cookies/quick bread/pastry/pie, each provided 7.2% of total energy intake; soft drinks/soda (5.4%) was the third highest ranked food source. The same three food groups, cake/cookies/quick bread/pastry/pie, yeast breads/rolls, and soft-drinks/soda were the highest ranked sources of energy also among the subgroup of adults 19–50 y (providing 6.7%, 6.6% and 6.6% of energy, respectively); however, among adults 51+ y, yeast breads/rolls (8.4%), cake/cookies/quick bread/pastry/pie (8.2%), and beef (4.8%) were the three highest ranked food sources of energy (Table 2). Poultry, beef, and cheese were the three highest ranked food sources of protein providing 14.4%, 14.0% and 8.5%, respectively, among adults 19+ y, and 15.1%, 14.5% and 9.3%, respectively, among adults 19–50 y; however, among adults 51+ y, beef (13.0%), poultry (13.0%), and milk (7.4%) were the highest ranked sources of protein (Table 3). 

The five highest sources of total fat (Table 4) among all adults were other fats and oils (9.8%), cheese (8.8%), beef (7.9%), cakes/cookies/quick bread/pastry/pie (7.7%) and salad dressings/mayonnaise (7.3%). Highest ranked sources of SFA were cheese (18.0%), beef (9.4%), and other fats and oils (8.8%) among adults 19–50 y; and cheese (13.7%), other fats and oils (9.2%) and margarine/butter (8.7%) among adults 51+ y (Table 5). 

Highest ranked food sources of carbohydrate were soft drinks/soda (13.9%), yeast breads/rolls (10.0%), and cake/cookies/quick bread/pastry/pie (8.3%) among adults 19–50 y; and yeast breads/rolls (12.9%), cake/cookies/quick bread/pastry/pie (10.3%), and fruit (7.5%) among adults 51+ y (Table 6). Yeast breads/rolls (10.2%) was the highest ranked source of dietary fiber among adults 19–50 y, while fruit (13.4%) was the highest ranked fiber source among adults 51+ y (Table 7). 

### 3.2. Micronutrients

Sources of calcium, vitamin D and potassium, three nutrients of public health concern identified by the 2010 DGA [17], are summarized in Table 8, Table 9, Table 10, respectively. Sodium, a nutrient of potential overconsumption is presented in Table 11. Dietary sources of all other micronutrients are summarized in Supplemental Tables S5–S17. 

Milk was the highest ranked food source of calcium (22.5%; Table 8), vitamin D (45.1%; Table 9), and potassium (9.6%; Table 10) among adults 19+ y. Although cheese was the highest ranked source of calcium (23.9%) among adults 19–50 y, milk remained the highest ranked source of calcium (24.1%) among adults 51+ y. Milk was the highest ranked source of vitamin D (19–50 y: 45.8%, 51+ y: 43.9%) among both age subgroups. Milk was the highest ranked source of potassium (9.7%) among adults 19–50 y; however, coffee/tea/other nonalcoholic beverages was the highest ranked source of potassium (10.8%) among adults 51+ y. 

In both age groups, the four highest ranked sources of sodium were the same (Table 11). For those 19–50 y and 51+ y, these were salt (23% and 21.7%, respectively), yeast bread and rolls (8.0% and 10.1%, respectively), cheese (7.9% and 6.4%, respectively), and frankfurters/sausages/luncheon meats (6.6% and 6.4%, respectively).

**Table 2:** Food sources of energy among US adults (from NHANES 2003–2006).

Food group ^a^	19+ years ^b^			19–50 years ^c^			51+ years ^d^		
	Energy			Energy			Energy		
	(mean = 9247 kJ)			(mean = 10,079 kJ)			(mean = 7950 kJ)		
	Ranking	% Total	Cumulative %	Ranking	% Total	Cumulative %	Ranking	% Total	Cumulative %
Yeast breads and rolls	1	7.2	7.2	2	6.6	6.6	1	8.4	8.4
Cakes, cookies, quick bread, pastry, pie	2	7.2	14.4	1	6.7	13.3	2	8.2	16.6
Soft drinks, soda (includes diet)	3	5.4	19.8	3	6.6	19.9	13	3	19.6
Beef	4	5	24.8	5	5.1	25	3	4.8	24.4
Alcoholic beverages	5	4.7	29.5	4	5.4	30.4	11	3.4	27.8
Cheese	6	4.6	34.1	6	4.9	35.3	6	3.9	31.7
Poultry	7	4.3	38.4	7	4.6	39.9	8	3.7	35.4
Candy, sugars and sugary foods	8	4.3	42.7	9	4.4	44.3	4	4.1	39.5
Crackers, popcorn, pretzels, chips	9	4.3	47	8	4.5	48.8	7	3.9	43.4
Milk	10	3.8	50.8	11	3.7	52.5	5	4.1	47.5
Biscuits, corn bread, pancakes, tortillas	11	3.6	54.4	10	3.9	56.4	14	3	50.5
Other fats and oils	12	3.6	58	12	3.6	60	9	3.6	54.1
Frankfurters, sausages, luncheon meats	13	3	61	14	2.9	62.9	12	3	57.1
Potatoes (white)	14	2.9	63.9	13	3.1	66	19	2.7	59.8
Salad dressings, mayonnaise	15	2.7	66.6	16	2.6	68.6	15	3	62.8
Flour, bran, baking ingredients	16	2.4	69	15	2.7	71.3	25	1.7	64.5
Fruit	17	2.3	71.3	26	1.6	72.9	10	3.5	68
Milk desserts	18	2.2	73.5	20	1.9	74.8	17	2.9	70.9
Margarine and butter	19	2.2	75.7	21	1.8	76.6	16	2.9	73.8
Nuts, seeds (including butters, pastes)	20	2.1	77.8	22	1.8	78.4	18	2.8	76.6
Pork, ham, bacon	21	2.1	79.9	18	2	80.4	21	2.3	78.9

^a^ Food groups (*n* = 9) contributing at least 1% in descending order: Ready-to-eat cereal; Pasta; Fruit drinks and ades; Fruit juice; Rice, cooked grains; Eggs; Mixtures, mostly grain; Legumes; and Fish and shellfish; ^b^ Data are Day 1 intakes by adults aged 19+ years (*n* = 9490); ^c^ Data are Day 1 intakes by adults aged 19–50 years (*n* = 5429); ^d^ Data are Day 1 intakes by adults aged 51+ years (*n* = 4061).

**Table 3:** Food sources of protein among US adults (from NHANES 2003–2006).

Food group ^a^	19+ years ^b^			19–50 years ^c^			51+ years ^d^		
	Protein			Protein			Protein		
	(mean = 84.2 g)			(mean = 90.4 g)			(mean = 74.6 g)		
	Ranking	% Total	Cumulative %	Ranking	% Total	Cumulative %	Ranking	% Total	Cumulative %
Poultry	1	14.4	14.4	1	15.1	15.1	2	13.0	13.0
Beef	2	14.0	28.4	2	14.5	29.6	1	13.0	26.0
Cheese	3	8.5	36.9	3	9.3	38.9	5	7.1	33.1
Milk	4	6.9	43.8	4	6.6	45.5	3	7.4	40.5
Yeast breads and rolls	5	6.4	50.2	5	5.9	51.4	4	7.3	47.8
Pork, ham, bacon	6	5.7	55.9	6	5.6	57.0	6	6.1	53.9
Fish and shellfish	7	5.0	60.9	7	4.5	61.5	7	5.9	59.8
Frankfurters, sausages, luncheon meats	8	4.4	65.3	8	4.5	66.0	8	4.2	64.0
Eggs	9	3.2	68.5	9	2.8	68.8	9	3.8	67.8
Cakes, cookies, quick bread, pastry, pie	10	2.5	71.0	11	2.4	71.2	10	2.7	70.5
Biscuits, corn bread, pancakes, tortillas	11	2.3	73.3	10	2.6	73.8	13	1.8	72.3
Nuts, seeds (including butters, pastes)	12	2.1	75.4	16	1.9	75.7	11	2.7	75.0

^a^ Food groups (*n* = 8) contributing at least 1% in descending order: Legumes; Pasta; Crackers, popcorn, pretzels, chips; Flour, bran, baking ingredients; Mixtures, mostly grain; Potatoes (white); Ready-to-eat cereals; and Milk desserts; ^b^ Data are Day 1 intakes by adults aged 19+ years (*n* = 9490); ^c^ Data are Day 1 intakes by adults aged 19–50 years (*n* = 5429); ^d^ Data are Day 1 intakes by adults aged 51+ years (*n* = 4061).

**Table 4:** Food sources of total fat among US adults (from NHANES 2003–2006).

Food group ^a^	19+ years ^b^			19–50 years ^c^			51+ years ^d^		
	Total fat			Total fat			Total fat		
	(mean = 84.0 g)			(mean = 90.3 g)			(mean = 74.2 g)		
	Ranking	% Total	Cumulative %	Ranking	% Total	Cumulative %	Ranking	% Total	Cumulative %
Other fats and oils	1	9.8	9.8	1	10.0	10.0	1	9.4	9.4
Cheese	2	8.8	18.6	2	9.7	19.7	6	7.2	16.6
Beef	3	7.9	26.5	3	8.2	27.9	5	7.3	23.9
Cakes, cookies, quick bread, pastry, pie	4	7.7	34.2	4	7.3	35.2	2	8.6	32.5
Salad dressings, mayonnaise	5	7.3	41.5	5	7.2	42.4	4	7.5	40.0
Margarine and butter	6	6.4	47.9	9	5.3	47.7	3	8.4	48.4
Frankfurters, sausages, luncheon meats	7	6.3	54.2	6	6.3	54.0	7	6.3	54.7
Crackers, popcorn, pretzels, chips	8	5.7	59.9	7	6.2	60.2	9	4.8	59.5
Poultry	9	5.3	65.2	8	5.9	66.1	10	4.1	63.6
Nuts, seeds (including butters, pastes)	10	4.8	70.0	10	4.0	70.1	8	6.3	69.9
Milk	11	3.7	73.7	11	3.8	73.9	12	3.5	73.4
Pork, ham, bacon	12	3.3	77.0	12	3.3	77.2	11	3.5	76.9
Biscuits, corn bread, pancakes, tortillas	13	2.8	79.8	13	3.0	80.2	16	2.5	79.4
Yeast breads and rolls	14	2.7	82.5	15	2.5	82.7	15	3.0	82.4
Milk desserts	15	2.7	85.2	16	2.3	85.0	13	3.3	85.7
Potatoes (white)	16	2.5	87.7	14	3.0	88.0	18	1.6	87.3
Eggs	17	2.5	90.2	18	2.2	90.2	14	3.1	90.4
Candy, sugars and sugary foods	18	2.1	92.3	17	2.3	92.5	17	1.9	92.3

^a^ Food groups (*n* = 1) contributing at least 1% in descending order: Mixtures, mostly grain; ^b^ Data are Day 1 intakes by adults aged 19+ years (*n* = 9490); ^c^ Data are Day 1 intakes by adults aged 19–50 years (*n* = 5429); ^d^ Data are Day 1 intakes by adults aged 51+ years (*n* = 4061).

**Table 5:** Food sources of saturated fatty acids (SFA) among US adults (from NHANES 2003–2006).

Food group ^a^	19+ years ^b^			19–50 years ^c^			51+ years ^d^		
	SFA			SFA			SFA		
	(mean = 27.9 g)			(mean = 30.2 g)			(mean = 24.4 g)		
	Ranking	% Total	Cumulative %	Ranking	% Total	Cumulative %	Ranking	% Total	Cumulative %
Cheese	1	16.5	16.5	1	18.0	18.0	1	13.7	13.7
Beef	2	9.1	25.6	2	9.4	27.4	4	8.4	22.1
Other fats and oils	3	8.9	34.5	3	8.8	36.2	2	9.2	31.3
Milk	4	6.7	41.2	4	6.8	43.0	7	6.6	37.9
Frankfurters, sausages, luncheon meats	5	6.7	47.9	5	6.6	49.6	5	6.8	44.7
Margarine and butter	6	6.3	54.2	7	5.1	54.7	3	8.7	53.4
Cakes, cookies, quick bread, pastry, pie	7	6.2	60.4	6	5.9	60.6	6	6.7	60.1
Milk desserts	8	4.9	65.3	9	4.2	64.8	8	6.2	66.3
Poultry	9	4.2	69.5	8	4.6	69.4	11	3.4	69.7
Crackers, popcorn, pretzels, chips	10	3.7	73.2	10	4.0	73.4	12	3.2	72.9
Pork, ham, bacon	11	3.5	76.7	11	3.4	76.8	9	3.7	76.6
Salad dressings, mayonnaise	12	3.4	80.1	12	3.3	80.1	10	3.5	80.1
Candy, sugars and sugary foods	13	3.0	83.1	13	3.2	83.3	15	2.7	82.8
Eggs	14	2.4	85.5	16	2.1	85.4	14	2.9	85.7
Nuts, seeds [including butters, pastes)	15	2.4	87.9	17	2.0	87.4	13	3.0	88.7
Yeast breads and rolls	16	2.0	89.9	19	1.9	89.3	16	2.3	91.0
Cheese	1	16.5	16.5	1	18.0	18.0	1	13.7	13.7

^a^ Food groups (*n* = 3) contributing at least 1% in descending order: Biscuits, corn bread, pancakes, tortillas; Mixtures, mostly grain; and Potatoes (white); ^b^ Data are Day 1 intakes by adults aged 19+ years (*n* = 9490); ^c^ Data are Day 1 intakes by adults aged 19–50 years (*n* = 5429); ^d^ Data are Day 1 intakes by adults aged 51+ years (*n* = 4061).

**Table 6:** Food sources of carbohydrate among US adults (from NHANES 2003–2006).

Food group ^a^	19+ years ^b^			19–50 years ^c^			51+ years ^d^		
	Carbohydrate			Carbohydrate			Carbohydrate		
	(mean = 266 g)			(mean = 291 g)			(mean = 228 g)		
	Ranking	% Total	Cumulative %	Ranking	% Total	Cumulative %	Ranking	% Total	Cumulative %
Soft drinks, soda (includes diet)	1	11.4	11.4	1	13.9	13.9	5	6.4	6.4
Yeast breads and rolls	2	10.9	22.3	2	10.0	23.9	1	12.9	19.3
Cakes, cookies, quick bread, pastry, pie	3	8.9	31.2	3	8.3	32.2	2	10.3	29.6
Candy, sugars and sugary foods	4	7.5	38.7	4	7.6	39.8	4	7.2	36.8
Fruit	5	4.8	43.5	10	3.5	43.3	3	7.5	44.3
Biscuits, corn bread, pancakes, tortillas	6	4.8	48.3	5	5.2	48.5	9	3.8	48.1
Crackers, popcorn, pretzels, chips	7	4.5	52.8	7	4.6	53.1	7	4.2	52.3
Flour, bran, baking ingredients	8	4.2	57.0	6	4.7	57.8	14	3.0	55.3
Potatoes (white)	9	4.0	61.0	9	3.9	61.7	8	4.2	59.5
Fruit drinks and ades	10	3.7	64.7	8	4.3	66.0	16	2.4	61.9
Ready-to-eat cereal	11	3.5	68.2	14	3.1	69.1	6	4.3	66.2
Fruit juice	12	3.5	71.7	11	3.4	72.5	10	3.7	69.9
Milk	13	3.1	74.8	15	3.0	75.5	11	3.5	73.4
Pasta	14	3.1	77.9	12	3.1	78.6	13	3.1	76.5
Rice, cooked grains	15	2.9	80.8	13	3.1	81.7	15	2.5	79.0
Milk desserts	16	2.4	83.2	17	2.0	83.7	12	3.3	82.3
Alcoholic beverages	17	2.1	85.3	16	2.5	86.2	20	1.4	83.7

^a^ Food groups (*n* = 4) contributing at least 1% in descending order: Legumes; Tomatoes, tomato/vegetable juice; Other vegetables; and Mixtures, mostly grain; ^b^ Data are Day 1 intakes by adults aged 19+ years (*n* = 9490); ^c^ Data are Day 1 intakes by adults aged 19–50 years (*n* = 5429); ^d^ Data are Day 1 intakes by adults aged 51+ years (*n* = 4061).

**Table 7:** Food sources of dietary fiber among US adults (from NHANES 2003–2006).

Food group ^a^	19+ years ^b^			19–50 years ^c^			51+ years ^d^		
	Dietary Fiber			Dietary Fiber			Dietary Fiber		
	(mean = 27.9 g)			(mean = 30.2 g)			(mean = 24.4 g)		
	Ranking	% Total	Cumulative %	Ranking	% Total	Cumulative %	Ranking	% Total	Cumulative %
Yeast breads and rolls	1	10.9	10.9	1	10.2	10.2	2	12.0	12.0
Fruit	2	10.2	21.1	3	8.1	18.3	1	13.4	25.4
Legumes	3	8.0	29.1	2	8.8	27.1	4	6.7	32.1
Potatoes (white)	4	6.4	35.5	5	6.8	33.9	5	5.7	37.8
Biscuits, corn bread, pancakes, tortillas	5	5.9	41.4	4	7.2	41.1	11	3.8	41.6
Crackers, popcorn, pretzels, chips	6	5.7	47.1	6	6.4	47.5	8	4.7	46.3
Other vegetables	7	5.5	52.6	7	5.6	53.1	6	5.4	51.7
Ready-to-eat cereal	8	5.4	58.0	10	4.4	57.5	3	6.9	58.6
Cakes, cookies, quick bread, pastry, pie	9	5.0	63.0	8	4.9	62.4	7	5.1	63.7
Tomatoes, tomato/vegetable juice	10	4.6	67.6	9	4.9	67.3	10	4.1	67.8
Nuts, seeds (including butters, pastes)	11	3.8	71.4	13	3.4	70.7	9	4.5	72.3
Pasta	12	3.1	74.5	12	3.5	74.2	13	2.6	74.9
Flour, bran, baking ingredients	13	3.0	77.5	11	3.6	77.8	15	2.0	76.9
Corn, peas, lima beans	14	2.4	79.9	16	1.9	79.7	12	3.1	80.0

^a^ Food groups (*n* = 11) contributing at least 1% in descending order: Broccoli, spinach, greens; Candy, sugars and sugary foods; Lettuce; Carrots, sweet potatoes, winter squash; Mixtures, mostly grain; Hot breakfast cereal; String beans (green, yellow, wax); Milk desserts; Fruit juice; Condiments an sauces; and Rice, cooked grains; ^b^ Data are Day 1 intakes by adults aged 19+ years (*n* = 9490); ^c^ Data are Day 1 intakes by adults aged 19–50 years (*n* = 5429); ^d^ Data are Day 1 intakes by adults aged 51+ years (*n* = 4061).

**Table 8:** Food sources of calcium among US adults (from NHANES 2003–2006).

Food group ^a^	19+ years ^b^			19–50 years ^c^			51+ years ^d^		
	Calcium			Calcium			Calcium		
	(mean = 922 mg)			(mean = 985 mg)			(mean = 825 mg)		
	Ranking	% Total	Cumulative %	Ranking	% Total	Cumulative %	Ranking	% Total	Cumulative %
Milk	1	22.5	22.5	2	21.6	21.6	1	24.1	24.1
Cheese	2	21.6	44.1	1	23.9	45.5	2	17.3	41.4
Yeast breads and rolls	3	7.3	51.4	3	6.9	52.4	3	8.2	49.6
Coffee, tea, other, nonalcoholic bevg.	4	4.4	55.8	4	4.5	56.9	5	4.3	53.9
Biscuits, corn bread, pancakes, tortillas	5	3.6	59.4	5	3.8	60.7	8	3.2	57.1
Milk desserts	6	3.5	62.9	7	2.9	63.6	4	4.6	61.7
Fruit juice	7	3.1	66.0	6	3.0	66.6	6	3.4	65.1
Cakes, cookies, quick bread, pastry, pie	8	3.0	69.0	8	2.8	69.4	7	3.2	68.3
Ready-to-eat cereal	9	2.2	71.2	10	1.8	71.2	9	2.8	71.1
Milk drinks	10	2.0	73.2	9	2.2	73.4	11	1.7	72.8

^a^ Food groups (*n* = 13) contributing at least 1% in descending order: Yogurt; Crackers, popcorn, pretzels, chips; Mixtures, mostly grain; Soft drinks, soda (including diet); Other vegetables; Eggs; Tomatoes, tomato/vegetable juice; Legumes; Meal replacements/supplements; Candy, sugars and sugary foods; Fruit drinks and ades; Other fats and oils; and Fruit; ^b^ Data are Day 1 intakes by adults aged 19+ years (*n* = 9490); ^c^ Data are Day 1 intakes by adults aged 19–50 years (*n* = 5429); ^d^ Data are Day 1 intakes by adults aged 51+ years (*n* = 4061).

**Table 9:** Food sources of vitamin D among US adults (from NHANES 2003–2006).

Food group ^a^	19+ years ^b^			19–50 years ^c^			51+ years ^d^		
	Vitamin D			Vitamin D			Vitamin D		
	(mean = 922 mg)			(mean = 985 mg)			(mean = 825 mg)		
	Ranking	% Total	Cumulative %	Ranking	% Total	Cumulative %	Ranking	% Total	Cumulative %
Milk	1	45.1	45.1	1	45.8	45.8	1	43.9	43.9
Fish and shellfish	2	14.4	59.5	2	12.9	58.7	2	16.8	60.7
Eggs	3	5.4	64.9	4	5.1	63.8	3	6.0	66.7
Ready-to-eat cereal	4	5.4	70.3	3	5.3	69.1	4	5.6	72.3
Fruit juice	5	3.6	73.9	7	3.6	72.7	5	3.6	75.9
Pork, ham, bacon	6	3.6	77.5	6	3.7	76.4	6	3.4	79.3
Milk drinks	7	3.1	80.6	5	3.7	80.1	10	2.2	81.5
Frankfurters, sausages, luncheon meats	8	3.1	83.7	9	3.2	83.3	7	3.0	84.5
Cheese	9	2.9	86.6	8	3.4	86.7	9	2.2	86.7
Margarine and butter	10	2.2	88.8	11	1.9	88.6	8	2.7	89.4
Meal replacements/supplements	11	2.0	90.8	10	2.2	90.8	11	1.6	91.0

^a^ Food groups (*n* = 5) contributing at least 1% in descending order: Beef; Cake, cookies, quick bread, pastry, pie; Yogurt; Poultry; and Milk desserts; ^b^ Data are Day 1 intakes by adults aged 19+ years (*n* = 9490); ^c^ Data are Day 1 intakes by adults aged 19–50 years (*n* = 5429); ^d^ Data are Day 1 intakes by adults aged 51+ years (*n* = 4061).

**Table 10:** Food sources of potassium among US adults (from NHANES 2003–2006).

Food group ^a^	19+ years ^b^			19–50 years ^c^			51+ years ^d^		
	Potassium			Potassium			Potassium		
	(mean = 2734 mg)			(mean = 2783 mg)			(mean = 2659 mg)		
	Ranking	% Total	Cumulative %	Ranking	% Total	Cumulative %	Ranking	% Total	Cumulative %
Milk	1	9.6	9.6	1	9.7	9.7	2	9.5	9.5
Coffee, tea, other, nonalcoholic bevg.	2	8.4	18.0	2	7.0	16.7	1	10.8	20.3
Potatoes (white)	3	6.7	24.7	3	6.9	23.6	4	6.4	26.7
Tomatoes, tomato/vegetable juice	4	5.9	30.6	4	6.1	29.7	5	5.5	32.2
Fruit	5	5.8	36.4	7	4.5	34.2	3	8.0	40.2
Beef	6	5.2	41.6	5	5.7	39.9	7	4.4	44.6
Fruit juice	7	5.0	46.6	6	5.2	45.1	6	4.8	49.4
Poultry	8	3.9	50.5	8	4.4	49.5	9	3.2	52.6
Crackers, popcorn, pretzels, chips	9	3.6	54.1	9	4.1	53.6	11	2.8	55.4
Other vegetables	10	3.4	57.5	10	3.4	57.0	8	3.5	58.9
Yeast breads and rolls	11	2.8	60.3	14	2.6	59.6	10	3.1	62.0
Pork, ham, bacon	12	2.7	63.0	12	2.7	62.3	12	2.6	64.6
Legumes	13	2.6	65.6	11	2.8	65.1	14	2.3	66.9
Frankfurters, sausages, luncheon meats	14	2.2	67.8	15	2.3	67.4	18	1.9	68.8
Alcoholic beverages	15	2.2	70.0	13	2.7	70.1	20	1.4	70.2
Fish and shellfish	16	2.2	72.2	17	2.0	72.1	13	2.4	72.6
Cakes, cookies, quick bread, pastry, pie	17	2.1	74.3	16	2.1	74.2	17	2.1	74.7

^a^ Food groups (*n* = 12) contributing at least 1% in descending order: Nuts, seeds (including butters, pastes); Milk desserts; Cheese; Biscuits, corn bread, pancakes, tortillas; Condiments and sauces; Ready-to-eat cereals; Broccoli, spinach, greens; Lettuce; Candy, sugars and sugary foods; Soup, broth, bouillon; Eggs; and Carrots, sweet potatoes, winter squash; ^b^ Data are Day 1 intakes by adults aged 19+ years (*n* = 9490); ^c^ Data are Day 1 intakes by adults aged 19–50 years (*n* = 5429); ^d^ Data are Day 1 intakes by adults aged 51+ years (*n* = 4061).

**Table 11:** Food sources of sodium among US adults (from NHANES 2003–2006).

Food group ^a^	19+ years ^b^			19–50 years ^c^			51+ years ^d^		
	Sodium			Sodium			Sodium		
	(mean = 922 mg)			(mean = 985 mg)			(mean = 825 mg)		
	Ranking	% Total	Cumulative %	Ranking	% Total	Cumulative %	Ranking	% Total	Cumulative %
Salt	1	22.6	22.6	1	23.0	23.0	1	21.7	21.7
Yeast breads and rolls	2	8.7	31.3	2	8.0	31.0	2	10.1	31.8
Cheese	3	7.4	38.7	3	7.9	38.9	3	6.4	38.2
Frankfurters, sausages, luncheon meats	4	6.6	45.3	4	6.6	45.5	4	6.4	44.6
Condiments and sauces	5	5.2	50.5	5	5.7	51.2	6	4.4	49.0
Pork, ham, bacon	6	4.5	55.0	7	4.3	55.5	5	4.9	53.9
Biscuits, corn bread, pancakes, tortillas	7	4.1	59.1	6	4.4	59.9	10	3.5	57.4
Crackers, popcorn, pretzels, chips	8	4.0	63.1	8	4.2	64.1	9	3.7	61.1
Cake, cookies, quick bread, pastry, pie	9	3.4	66.5	9	3.1	67.2	8	3.8	64.9
Soup, broth, bouillon	10	3.2	69.7	11	2.8	70.0	7	3.9	68.8
Tomatoes, tomato/vegetable juice	11	3.0	72.7	10	2.8	72.8	11	3.2	72.0
Salad dressings, mayonnaise	12	2.7	75.4	12	2.6	75.4	12	2.9	74.9
Milk	13	2.1	77.5	14	2.0	77.4	13	2.3	77.2

^a^ Food groups (*n* = 11) contributing at least 1% in descending order: Poultry; Ready-to-eat cereal; Margarine and butter; Mixtures, mostly grain; Olives, pickles; Legumes; Beef; Potatoes (white); Fish and shellfish; Other fats and oils; and Coffee, tea, other, nonalcoholic beverages; ^b^ Data are Day 1 intakes by adults aged 19+ years (*n* = 9490); ^c^ Data are Day 1 intakes by adults aged 19–50 years (*n* = 5429); ^d^ Data are Day 1 intakes by adults aged 51+ years (*n* = 4061).

## 4. Discussion

Subar *et al.* [30] and Cotton *et al.* [31] ranked food sources of energy and nutrients among adults using CSFII data from 1989 to 1991 and 1994 to 1996, respectively. These studies were conducted with data that is now at least 15 years old. Since then, influences in the diets of adults have included food product reformulations in response to consumers’ health concerns [42], food availability and cost [43,44], changing food and beverage preferences [45,46]; increased consumption of meals away from home [47]; and trade liberalization, which has increased the number and types of foods available throughout the year and throughout wider geographic regions [48]. Thus, it was important to re-assess the diets of adults using recent nationally representative data. While both previous studies looked at all adult age groups combined, the five highest ranked sources of energy (for 1994–1996 data) were: yeast bread (8.7%), beef (6.2%), cakes/cookies/quick breads/doughnuts (5.7%), soft drinks/soda (5.2%), and milk (4.2%), which provided approximately 30% of total energy. Compared to the current work, yeast bread (8.7%) and milk (3.8%) were a lower percentage of energy while cakes/cookies/quick breads/doughnuts (7.2%) were now a higher percentage. In our study alcoholic beverages (4.7%) made it into the top five energy sources (was eighth at 3.3% in previous work [31]). Overall, both the current and previous studies showed energy-dense, nutrient-poor foods (e.g., cakes/cookies/quick breads/doughnuts, soft drinks/soda, *etc.*) as major contributors to dietary energy intake, making it difficult for individuals to achieve nutrient requirements without exceeding energy limits.

The five highest ranked food sources of energy consumed by adults 19–50 y were considerably different from those ranked highest among adults 51+ y. Among adults 19–50 y, the five highest ranked sources of energy were yeast breads/rolls, cake/cookies/quick bread/pastry/pie, soft drinks/soda, beef, and alcoholic beverages. Among those aged 51+ y, soft drinks/soda and alcoholic beverages ranked below the five highest ranked foods; candy/sugars/sugary food and milk were among the five highest ranked sources of energy. 

Yeast breads/rolls, especially if whole grain; beef, if lean; and milk were the most nutrient-dense food sources of energy; together, they provided protein, MUFA, PUFA, iron, zinc, calcium, vitamin D, and potassium (some data not shown). The other energy sources provided total sugars, fats, and few micronutrients. 

Consistent with these findings, the 2010 DGA indicated the highest ranking food energy source in the US adult population was grain-based desserts (cakes/cookies/doughnuts/pies/crisps/cobblers/granola bars), which contributed 582 kJ/day; and the highest ranking beverage energy source was soda, which contributed 477 kJ/day [17]. The total of 1059 kJ provides 95% of the discretionary calories from these two food groups alone for an individual with an 8368 kJ diet [49]. Meeting nutrient intake recommendations while staying within energy needs has proven to be challenging for many Americans [5], and as a result, individuals may be overweight or obese while being undernourished. To help Americans improve dietary habits and meet nutrient needs through foods, understanding food sources of nutrients is critical. 

In contrast to the highest ranked food sources of energy, the highest ranked food sources of protein were more nutrient-dense foods: poultry, beef, cheese, milk, and yeast bread/rolls. These foods contributed approximately 50% of protein intake; although the rank order was different, the same five sources were reported by Subar *et al.* [30] and Cotton *et al.* [31]. Compared with the earlier studies where beef was the first highest ranked food source of protein; in this study, poultry was the first highest ranked food source of protein among all adults and those 19–50 y. Changes in ranking of beef, poultry, and cheese are consistent with temporal trends in intake [3,50]. These foods were also good to excellent sources of B vitamins, vitamin D, calcium, potassium, iron, or zinc; and, when consumed in their lowest fat form, they are consistent with foods recommended by MyPlate [49]. 

The 2010 DGA recognized low intakes of dietary fiber, calcium, vitamin D, and potassium were of public health concern [25]. Ideally, foods containing a constellation of these micronutrients can be selected without unduly increasing energy intake. If the food groups consumed most frequently are not those most nutrient dense, reformed public policies and better nutrition education programs are needed. 

Dietary fiber intake by adults is slightly less than half the recommended amount [16]. Yeast breads/rolls, legumes, and fruit were the three highest ranked food sources of fiber among adults 19–50 y. Among adults 51 y and older, ready-to-eat cereal (RTEC) ranked third and legumes ranked fourth. All of these foods are nutrient dense, and contributed to dietary intakes of B vitamins, vitamin C, folate, or potassium, in addition to fiber. Consumption of legumes [51] and fruit [52] is associated with improved diet quality. Although these foods are nutrient dense and improve diet quality, most Americans do not consume the recommended amount of or legumes [51] or fruit [5,6]. The highest ranked food sources of dietary fiber shown previously [30,31] were yeast breads, dried beans/lentils, potatoes (white), RTEC, and tomatoes. A comparison of the two studies [30,31] with the present one suggests a shift among foods that contribute to fiber intake occurred over time. In this study, vegetables were split into many different categories, however, when the vegetables that were the highest ranked sources of dietary fiber, *i.e.*, potatoes (white) (6.4%), other vegetables (5.5%), tomatoes/tomato/vegetable juice (4.6%), were combined with categories contributing at least 1% of fiber (e.g., broccoli, spinach, greens; lettuce; carrots, sweet potatoes, winter squash; string beans), vegetables would be the highest ranked source, providing at least 20% of fiber intake.

The nutrient contribution of milk and dairy products play an important role in helping Americans meet recommendations for short-fall nutrients [53] and nutrients of public health concern [25]. In our study, milk was an important source of calcium, potassium, vitamin D, and many other nutrients, including vitamin A, thiamin, riboflavin, vitamins B6 and B12, phosphorus, magnesium, and zinc (some data not shown). Subar *et al.* [30] showed that in 1989–1991, milk provided 34.2% of calcium intake, and Cotton *et al.* [31] showed that in 1994–1996, 28.3% of calcium was supplied by milk. In our study milk provided only 22.5% of calcium intake and there were a larger number of smaller contributors to calcium intake than seen in previous of studies, primarily due to fortification of various products (e.g., fruit juice, RTEC).

This study had several limitations. Food grouping has a major influence on the ranked order of food groups; thus, caution is advised when comparing these data to previous reports if there were differences in the level of aggregation or disaggregation procedures used to include ingredients in food groups. Study outcomes are based on self-reported data which may underestimate energy intake [54]. A single day’s intake may not necessarily be representative of usual intake; however, the mean of the intake distribution drawn from a large, representative sample of a group is not affected by day-to-day variation [55]. Since the contribution of food sources in this study was based on mean intake data, the use of a single 24-h recall was appropriate [56]. Since the food grouping in the DSN database does not include ingredients of manufactured foods, disaggregated foods represent mixtures that are prepared from recipes. Salt was separated from other ingredients of disaggregated foods. The USDA reduces the sodium content of mixtures if the respondent never, rarely, or occasionally uses salt in cooking, and the food was prepared at home; thus, much of the salt came from disaggregated foods prepared by restaurants, schools, and other establishments. The updated vitamin D database was appropriate for use with the NHANES 2005–2006 dietary intake data; however, since vitamin D intake from foods consumed in 2003–2004 was determined using the updated food composition data, the 2003–2004 intake data may not have been representative of that time period. Any variation in food composition data affects the reliability of dietary intake estimation. Fortified foods provide the major sources of vitamin D in the diet; vitamin D fortification is regulated by a food additive rule [57]. A petition to add vitamin D to calcium-supplemented juices and fruit drinks was approved by the Food and Drug Administration and implemented in 2004 [58]; however, this change was effective only in the NHANES 2005–2006, since the new calcium- and vitamin D-supplemented foods were not reported in the NHANES 2003–2004. 

## 5. Conclusions

In conclusion, this study showed that adults consumed a large proportion of total energy from energy-dense, low-nutrient food groups, and identified principal sources of energy that were also major sources of nutrients. While some food and beverage choices such as milk, fruit juices, lean beef and poultry are nutrient-dense and food sources of many nutrients, other energy sources such as cakes/cookies/pies and soda/soft drinks are energy-dense and nutrient-poor choices. All health professionals, policy makers, and the nutrition community need to be aware of the types of foods Americans are consuming since this knowledge can help health professionals design effective strategies to improve diet quality, and reduce energy consumption by US adults.

## Supplementary Files

Supplementary File 1 Supplemental Tables (PDF, 419 KB)
