# Supplementary material for: Food Sources of Energy and Nutrients among Adults in the US: NHANES 2003–2006
Source: Nutrients. 2012 Dec 19;4(12):2097–120. doi: 10.3390/nu4122097 (PMC3546624; doi:10.3390/nu4122097)
Supplement: Supplementary File 1 — Supplemental Tables (PDF, 419 KB) [file nutrients-04-02097-s001.pdf]

Supplemental Tables

Table S1. Food sources of monounsaturated fatty acids (MUFA) among US adults (from NHANES 2003–2006). 2
Table S2. Food sources of polyunsaturated fatty acids (PUFA) among US adults (from NHANES 2003–2006). 3
Table S3. Food sources of cholesterol among US adults (from NHANES 2003–2006). 4
Table S4. Food sources of total sugars among US adults (from NHANES 2003–2006). 5
Table S5. Food sources of vitamin A (retinoic acid equivalents; RAE) among US adults (from NHANES 2003–2006). 6
Table S6. Food sources of vitamin E (alpha-tocopherol; AT) among US adults (from NHANES 2003–2006). 7
Table S7. Food sources of vitamin C among US adults (from NHANES 2003–2006). 8
Table S8. Food sources of thiamin among US adults (from NHANES 2003–2006). 9
Table S9. Food sources of riboflavin among US adults (from NHANES 2003–2006). 10
Table S10. Food sources of niacin among US adults (from NHANES 2003–2006). 11
Table S11. Food sources of vitamin B_6 among US adults (from NHANES 2003–2006). 12
Table S12. Food sources of folate (dietary folate equivalents; DFE) among US adults (from NHANES 2003–2006). 13
Table S13. Food sources of vitamin B_{12} among US adults (from NHANES 2003–2006). 14
Table S14. Food sources of phosphorus among US adults (from NHANES 2003–2006). 15
Table S15. Food sources of magnesium among US adults (from NHANES 2003–2006). 16
Table S16. Food sources of iron among US adults (from NHANES 2003–2006). 17
Table S17. Food sources of zinc among US adults (from NHANES 2003–2006). 18

Table S1. Food sources of monounsaturated fatty acids (MUFA) among US adults (from NHANES 2003–2006).

Food group [a]	19+ years [b] MUFA (mean = 31.3 g)			19–50 years [c] MUFA (mean = 33.7 g)			51+ years [d] MUFA (mean = 27.5 g)		
	Ranking	% Total	Cumulative %	Ranking	% Total	Cumulative %	Ranking	% Total	Cumulative %
Other fats and oils	1	10.5	10.5	1	10.9	10.9	1	9.9	9.9
Beef	2	9.2	19.7	2	9.5	20.4	3	8.6	18.5
Cakes, cookies, quick bread, pastry, pie	3	8.9	28.6	3	8.4	28.8	1	9.9	28.4
Frankfurters, sausages, luncheon meats	4	7.5	36.1	4	7.4	36.2	6	7.6	36.0
Cheese	5	6.6	42.7	5	7.3	43.5	7	5.5	41.5
Margarine and butter	6	6.4	49.1	7	5.4	48.9	4	8.3	49.8
Nuts, seeds (including butters, pastes)	7	6.2	55.3	10	5.1	54.0	5	8.2	58.0
Poultry	8	5.5	60.8	6	6.2	60.2	10	4.3	62.3
Salad dressings, mayonnaise	9	5.2	66.0	9	5.2	65.4	8	5.4	67.7
Crackers, popcorn, pretzels, chips	10	4.9	70.9	8	5.3	70.7	9	4.3	72.0
Pork, ham, bacon	11	4.0	74.9	12	3.9	74.6	11	4.3	76.3
Potatoes (white)	12	3.7	78.6	11	4.5	79.1	17	2.3	78.6
Biscuits, corn bread, pancakes, tortillas	13	3.4	82.0	13	3.7	82.8	13	2.8	81.4
Milk	14	2.6	84.6	14	2.7	85.5	14	2.5	83.9
Eggs	15	2.5	87.1	15	2.2	87.7	12	3.0	86.9
Yeast breads and rolls	16	2.1	89.2	17	1.9	89.6	16	2.3	89.2
Candy, sugars and sugary foods	17	2.1	91.3	16	2.2	91.8	18	1.8	91.0
Milk desserts	18	2.0	93.3	19	1.7	93.5	15	2.4	93.4

[a] Food groups (n = 1) contributing at least 1% in descending order: Mixtures, mostly grain; [b] Data are Day 1 intakes by adults aged 19+ years (n = 9490); [c] Data are Day 1 intakes by adults aged 19–50 years (n = 5429); [d] Data are Day 1 intakes by adults aged 51+ years (n = 4061).

Table S2. Food sources of polyunsaturated fatty acids (PUFA) among US adults (from NHANES 2003–2006).

**Food group** [a]	**19+ years** [b]			**19–50 years** [c]			**51+ years** [d]		
	**PUFA (mean = 17.7 g)**			**PUFA (mean = 18.8 g)**			**PUFA (mean = 16.0 g)**		
	**Ranking**	**% Total**	**Cumulative %**	**Ranking**	**% Total**	**Cumulative %**	**Ranking**	**% Total**	**Cumulative %**
Salad dressings, mayonnaise	1	17.8	17.8	1	17.7	17.7	1	17.9	17.9
Other fats and oils	2	11.7	29.5	2	12.4	30.1	2	10.4	28.3
Crackers, popcorn, pretzels, chips	3	9.6	39.1	3	10.6	40.7	6	7.7	36.0
Cakes, cookies, quick bread, pastry, pie	4	8.9	48.0	4	8.3	49.0	3	10.0	46.0
Margarine and butter	5	7.4	55.4	6	6.2	55.2	4	9.4	55.4
Nuts, seeds (including butters, pastes)	6	7.1	62.5	7	6.0	61.2	5	9.1	64.5
Poultry	7	5.8	68.3	5	6.6	67.8	8	4.5	69.0
Yeast breads and rolls	8	4.6	72.9	8	4.4	72.2	7	5.0	74.0
Biscuits, corn bread, pancakes, tortillas	9	3.6	76.5	9	3.8	76.0	9	3.2	77.2
Frankfurters, sausages, luncheon meats	10	3.2	79.7	10	3.3	79.3	10	3.0	80.2
Potatoes (white)	11	2.2	81.9	11	2.6	81.9	13	1.5	81.7

[a] Food groups (n = 7) contributing at least 1% in descending order: Eggs; Pork, ham, bacon; Cheese; Beef; Mixtures, mostly grain; Candy, sugars and sugary foods; and Fish and shellfish; [b] Data are Day 1 intakes by adults aged 19+ years (n = 9490); [c] Data are Day 1 intakes by adults aged 19–50 years (n = 5429); [d] Data are Day 1 intakes by adults aged 51+ years (n = 4061).

Table S3. Food sources of cholesterol among US adults (from NHANES 2003–2006).

**Food group** [a]	**19+ years** [b]			**19–50 years** [c]			**51+ years** [d]		
	**Cholesterol (mean = 291 mg)**			**Cholesterol (mean = 304 mg)**			**Cholesterol (mean = 272 mg)**		
	**Ranking**	**% Total**	**Cumulative %**	**Ranking**	**% Total**	**Cumulative %**	**Ranking**	**% Total**	**Cumulative %**
Eggs	1	29.7	29.7	1	27.1	27.1	1	34.3	34.3
Poultry	2	13.1	41.0	2	14.3	41.4	3	11.1	45.4
Beef	3	12.7	53.7	3	13.5	54.9	2	11.3	56.7
Cheese	4	7.9	61.6	4	8.9	63.8	4	6.2	62.9
Fish and shellfish	5	5.3	66.9	6	5.1	68.9	5	5.7	68.6
Frankfurters, sausages, luncheon meats	6	5.3	72.2	5	5.5	74.4	7	4.9	73.5
Pork, ham, bacon	7	5.0	77.2	7	5.0	79.4	6	5.0	78.5
Milk	8	4.1	81.3	8	4.2	83.6	9	3.9	82.4
Cakes, cookies, quick bread, pastry, pie	9	3.9	85.2	9	3.7	87.3	8	4.2	86.6
Milk desserts	10	2.8	88.0	10	2.6	89.9	10	3.3	89.9

[a] Food groups (*n* = 4) contributing at least 1% in descending order: Other fats and oils; Margarine and butter; Biscuits, corn bread, pancakes, tortillas; and Mixtures, mostly grain; [b] Data are Day 1 intakes by adults aged 19+ years (*n* = 9490); [c] Data are Day 1 intakes by adults aged 19–50 years (*n* = 5429); [d] Data are Day 1 intakes by adults aged 51+ years (*n* = 4061).

Table S4. Food sources of total sugars among US adults (from NHANES 2003–2006).

**Food group** [a]	**19+ years** [b]			**19–50 years** [c]			**51+ years** [d]		
	**Total Sugars (mean = 291 mg)**			**Total Sugars (mean = 304 mg)**			**Total Sugars (mean = 272 mg)**		
	**Ranking**	**% Total**	**Cumulative %**	**Ranking**	**% Total**	**Cumulative %**	**Ranking**	**% Total**	**Cumulative %**
Soft drinks, soda (includes diet)	1	22.7	22.7	1	27.4	27.4	2	13.0	13.0
Candy, sugars and sugary foods	2	13.6	36.3	2	13.6	41.0	1	13.6	26.6
Cakes, cookies, quick bread, pastry, pie	3	11.0	47.3	3	10.0	51.0	3	12.9	39.5
Fruit	4	7.4	54.7	7	5.3	56.3	4	11.8	51.3
Milk	5	7.3	62.0	5	6.8	63.1	5	8.4	59.7
Fruit drinks and ades	6	7.0	69.0	4	8.1	71.2	8	4.7	64.4
Fruit juice	7	6.7	75.7	6	6.4	77.6	6	7.2	71.6
Milk desserts	8	4.3	80.0	8	3.6	81.2	7	5.8	77.4
Yeast breads and rolls	9	2.3	82.3	10	2.0	83.2	9	2.8	80.2
Ready-to-eat cereal	10	2.2	84.5	9	2.2	85.4	10	2.3	82.5

[a] Food groups (n = 4) contributing at least 1% in descending order: Tomatoes, tomato/vegetable juice; Milk drinks; Condiments and sauces; and Other vegetables; [b] Data are Day 1 intakes by adults aged 19+ years (n = 9490); [c] Data are Day 1 intakes by adults aged 19–50 years (n = 5429); [d] Data are Day 1 intakes by adults aged 51+ years (n = 4061).

Table S5. Food sources of vitamin A (retinoic acid equivalents; RAE) among US adults (from NHANES 2003–2006).

Food group [a]	19+ years [b] Vitamin A (mean = 615 µg RAE)			19–50 years [c] Vitamin A (mean = 605 µg RAE)			51+ years [d] Vitamin A (mean = 631 µg RAE)		
	Ranking	% Total	Cumulative %	Ranking	% Total	Cumulative %	Ranking	% Total	Cumulative %
Milk	1	13.9	13.9	1	14.2	14.2	1	13.5	13.5
Carrots, sweet potatoes, winter squash	2	11.6	25.5	2	11.2	25.4	2	12.3	25.8
Cheese	3	9.2	34.7	3	10.9	36.3	5	6.6	19.2
Margarine and butter	4	8.9	43.6	5	7.8	44.1	3	10.4	29.6
Ready-to-eat cereal	5	8.2	51.8	4	8.2	52.3	4	8.2	37.8
Cakes, cookies, quick bread, pastry, pie	6	4.7	56.5	6	4.9	57.2	7	4.5	42.3
Eggs	7	4.6	61.1	7	4.4	61.6	6	4.8	47.1
Broccoli, spinach, greens	8	3.9	65.0	8	3.6	65.2	8	4.4	51.5
Milk desserts	9	3.7	68.7	10	3.5	68.7	9	4.1	55.6
Organ meats	10	3.7	72.4	9	3.5	72.2	10	3.9	59.5
Tomatoes, tomato/vegetable juice	11	3.2	75.6	11	3.2	75.4	11	3.3	62.8
Other fats and oils	12	2.4	78.0	12	2.5	77.9	14	2.2	65.0
Fruit	13	2.0	80.0	17	1.4	79.3	12	2.9	67.9

[a] Food groups (*n* = 6) contributing at least 1% in descending order: Hot breakfast cereal; Meal replacements/supplements; Milk drinks; Lettuce; Biscuits, corn bread, pancakes, tortillas; and Poultry; [b] Data are Day 1 intakes by adults aged 19+ years (*n* = 9490); [c] Data are Day 1 intakes by adults aged 19–50 years (*n* = 5429); [d] Data are Day 1 intakes by adults aged 51+ years (*n* = 4061).

Table S6. Food sources of vitamin E (alpha-tocopherol; AT) among US adults (from NHANES 2003–2006).

**Food group [a]**	**19+ years [b]**			**19–50 years [c]**			**51+ years [d]**		
	**Vitamin E (mean = 7.3 mg AT)**			**Vitamin E (mean = 7.6 mg AT)**			**Vitamin E (mean = 6.8 mg AT)**		
	**Ranking**	**% Total**	**Cumulative %**	**Ranking**	**% Total**	**Cumulative %**	**Ranking**	**% Total**	**Cumulative %**
Nuts, seeds (including butters, pastes)	1	10.0	10.0	3	9.2	9.2	1	11.3	11.3
Tomatoes, tomato/vegetable juice	2	9.0	19.0	1	9.4	18.6	3	8.3	19.6
Crackers, popcorn, pretzels, chips	3	8.2	27.2	2	9.2	27.8	6	6.4	26.0
Salad dressings, mayonnaise	4	8.1	35.3	4	8.2	36.0	4	7.9	33.9
Margarine and butter	5	6.8	42.1	7	5.7	41.7	2	8.9	42.8
Cake, cookies, quick bread, pastry, pie	6	6.7	48.8	5	6.7	48.4	5	6.6	49.4
Other fats and oils	7	5.8	54.6	6	6.2	54.6	8	5.1	54.5
Ready-to-eat cereal	8	4.0	58.6	8	3.3	57.9	7	5.3	59.8
Eggs	9	2.8	61.4	10	2.5	60.4	10	3.3	63.1
Poultry	10	2.8	64.2	9	3.2	63.6	14	2.0	65.1
Fruit	11	2.4	66.6	16	1.8	65.4	9	3.5	68.6
Fish and shellfish	12	2.4	69.0	13	2.2	67.6	11	2.8	71.4
Beef	13	2.3	71.3	11	2.5	70.1	15	2.0	73.4
Meal replacements/supplements	14	2.1	73.4	12	2.3	72.4	17	1.7	75.1
Broccoli, spinach, greens	15	2.1	75.5	14	1.9	74.3	12	2.3	77.4

[a] Food groups (*n* = 12) contributing at least 1% in descending order: Yeast breads and rolls; Biscuits, corn bread, pancakes, tortillas; Fruit juice; Other vegetables; Condiments and sauces; Candy, sugars and sugary foods; Coffee, tea, other, nonalcoholic beverages.; Potatoes (white); Cheese; Legumes; Carrots, sweet potatoes, winter squash; and Milk desserts; [b] Data are Day 1 intakes by adults aged 19+ years (*n* = 9490); [c] Data are Day 1 intakes by adults aged 19–50 years (*n* = 5429); [d] Data are Day 1 intakes by adults aged 51+ years (*n* = 4061).

Table S7. Food sources of vitamin C among US adults (from NHANES 2003–2006).

**Food group** [a]	**19+ years** [b]			**19–50 years** [c]			**51+ years** [d]		
	**Vitamin C (mean = 87.7 mg)**			**Vitamin C (mean = 89.1 mg)**			**Vitamin C (mean = 85.4 mg)**		
	**Ranking**	**% Total**	**Cumulative %**	**Ranking**	**% Total**	**Cumulative %**	**Ranking**	**% Total**	**Cumulative %**
Fruit juice	1	28.6	28.6	1	29.4	29.4	1	27.2	27.2
Fruit	2	15.3	43.9	3	12.3	41.7	2	20.1	47.3
Fruit drinks and ades	3	14.0	57.9	2	16.5	58.2	4	10.0	57.3
Other vegetables	4	11.3	69.2	4	11.6	69.8	3	10.8	68.1
Tomatoes, tomato/vegetable juice	5	7.2	76.4	5	7.1	76.9	5	7.3	75.4
Broccoli, spinach, greens	6	5.1	81.5	6	4.9	81.8	6	5.4	80.8
Potatoes (white)	7	4.5	86.0	7	4.2	86.0	7	5.1	85.9
Ready-to-eat cereals	8	2.4	88.4	8	2.1	88.1	8	3.0	88.9

[a] Food groups (*n* = 4) contributing at least 1% in descending order: Condiments and sauces; Cake, cookies, quick bread, pastry, pie; Corn, peas, lima beans; and Lettuce; [b] Data are Day 1 intakes by adults aged 19+ years (*n* = 9490); [c] Data are Day 1 intakes by adults aged 19–50 years (*n* = 5429); [d] Data are Day 1 intakes by adults aged 51+ years (*n* = 4061).

Table S8. Food sources of thiamin among US adults (from NHANES 2003–2006).

Food group [a]	19+ years [b] Thiamin (mean = 1.7 mg)			19–50 years [c] Thiamin (mean = 1.8 mg)			51+ years [d] Thiamin (mean = 1.6 mg)		
	**Ranking**	**% Total**	**Cumulative %**	**Ranking**	**% Total**	**Cumulative %**	**Ranking**	**% Total**	**Cumulative %**
Yeast breads and rolls	1	14.7	14.7	1	14.1	14.1	1	15.8	15.8
Ready-to-eat cereal	2	9.9	24.6	2	8.8	22.9	2	11.8	27.6
Pork, ham, bacon	3	8.0	32.6	3	7.9	30.8	3	8.1	35.7
Cake, cookies, quick bread, pastry, pie	4	6.2	38.8	6	6.5	37.3	4	5.8	41.5
Biscuits, corn bread, pancakes, tortillas	5	5.7	44.5	4	6.6	43.9	5	4.3	45.8
Flour, bran, baking ingredients	6	5.5	50.0	5	6.5	50.4	8	3.6	49.4
Pasta	7	4.3	54.3	7	4.4	54.8	7	4.0	53.4
Milk	8	4.0	58.3	8	4.0	58.8	6	4.1	57.5
Potatoes (white)	9	3.0	61.3	9	3.0	61.8	10	3.0	60.5
Frankfurters, sausages, luncheon meats	10	2.9	64.2	10	3.0	64.8	11	2.7	63.2
Fruit juice	11	2.6	66.8	12	2.6	67.4	12	2.7	65.9
Rice, cooked grains	12	2.5	69.3	11	2.8	70.2	16	2.0	67.9
Coffee, tea, other, nonalcoholic bevg.	13	2.3	71.6	17	1.9	72.1	9	3.0	70.9
Crackers, popcorn, pretzels, chips	14	2.2	73.8	13	2.3	74.4	14	2.1	73.0
Legumes	15	2.1	75.9	14	2.1	76.5	15	2.1	75.1
Yeast breads and rolls	1	14.7	14.7	1	14.1	14.1	1	15.8	15.8

[a] Food groups (n = 11) contributing at least 1% in descending order: Poultry; Fruit; Mixtures, mostly grain; Beef; Meal replacements/supplements; Other vegetables; Tomatoes, tomato vegetable juice; Hot breakfast cereal; Nuts, seeds (including butters, pastes); Fish and shellfish; and Corn, peas, lima beans; [b] Data are Day 1 intakes by adults aged 19+ years (n = 9490); [c] Data are Day 1 intakes by adults aged 19–50 years (n = 5429); [d] Data are Day 1 intakes by adults aged 51+ years (n = 4061).

Table S9. Food sources of riboflavin among US adults (from NHANES 2003–2006).

Food group [a]	19+ years [b] Riboflavin (mean = 2.3 mg)			19–50 years [c] Riboflavin (mean = 2.4 mg)			51+ years [d] Riboflavin (mean = 2.2 mg)		
	**Ranking**	**% Total**	**Cumulative %**	**Ranking**	**% Total**	**Cumulative %**	**Ranking**	**% Total**	**Cumulative %**
Milk	1	14.1	14.1	1	14.1	14.1	1	14.2	14.2
Coffee, tea, other, nonalcoholic bevg.	2	10.2	24.3	2	8.6	22.7	2	12.8	27.0
Yeast breads and rolls	3	7.9	32.2	3	7.7	30.4	4	8.4	35.4
Ready-to-eat cereal	4	7.9	40.1	4	7.2	37.6	3	9.1	44.5
Cheese	5	5.2	45.3	5	5.8	43.4	6	4.1	48.6
Eggs	6	4.3	49.6	7	3.9	47.3	5	4.9	53.5
Cake, cookies, quick bread, pastry, pie	7	4.3	53.9	6	4.4	51.7	7	4.1	57.6
Beef	8	3.6	57.5	8	3.8	55.5	8	3.2	60.8
Biscuits, corn bread, pancakes, tortillas	9	3.3	60.8	9	3.6	59.1	10	2.8	63.6
Poultry	10	3.1	63.9	11	3.4	62.5	11	2.6	66.2
Flour, bran, baking ingredients	11	2.9	66.8	10	3.5	66.0	14	1.9	68.1
Milk desserts	12	2.4	69.2	16	2.0	68.0	9	2.9	71.0
Pork, ham, bacon	13	2.3	71.5	12	2.4	70.4	12	2.3	73.3
Crackers, popcorn, pretzels, chips	14	2.1	73.6	13	2.3	72.7	16	1.8	75.1
Frankfurters, sausages, luncheon meats	15	2.1	75.7	15	2.2	74.9	15	1.8	76.9

[a] Food groups (*n* = 10) contributing at least 1% in descending order: Alcoholic beverages; Milk drinks; Pasta; Fruit; Soft drinks, soda (including diet); Other vegetables; Tomatoes, tomato/vegetable juice; Mixtures, mostly grain; Fruit drinks and ades; and Meal replacements/supplements; [b] Data are Day 1 intakes by adults aged 19+ years (*n* = 9490); [c] Data are Day 1 intakes by adults aged 19–50 years (*n* = 5429); [d] Data are Day 1 intakes by adults aged 51+ years (*n* = 4061).

Table S10. Food sources of niacin among US adults (from NHANES 2003–2006).

Food group [a]	19+ years [b] Niacin (mean = 25.2 mg)			19–50 years [c] Niacin (mean = 27.1 mg)			51+ years [d] Niacin (mean = 22.4 mg)		
	Ranking	% Total	Cumulative %	Ranking	% Total	Cumulative %	Ranking	% Total	Cumulative %
Poultry	1	15.4	15.4	1	16.2	16.2	1	13.9	13.9
Yeast breads and rolls	2	9.9	25.3	3	9.2	25.4	2	11.2	25.1
Beef	3	9.2	34.5	2	9.6	35.0	4	8.4	33.5
Ready-to-eat cereal	4	8.0	42.5	4	7.0	42.0	3	9.9	43.4
Pork, ham, bacon	5	4.3	46.8	5	4.2	46.2	6	4.6	48.0
Coffee, tea, other, nonalcoholic bevg.	6	3.9	50.7	12	3.5	49.7	5	4.7	52.7
Fish and shellfish	7	3.9	54.6	10	3.6	53.3	7	4.5	57.2
Frankfurters, sausages, luncheon meats	8	3.6	58.2	7	3.8	57.1	10	3.4	60.6
Cake, cookies, quick bread, pastry, pie	9	3.6	61.8	8	3.6	60.7	9	3.5	64.1
Alcoholic beverages	10	3.3	65.1	6	4.1	64.8	17	1.8	65.9
Biscuits, corn bread, pancakes, tortillas	11	3.3	68.4	11	3.5	68.3	13	2.7	68.6
Flour, bran, baking ingredients	12	3.1	71.5	9	3.6	71.9	15	2.1	70.7
Nuts, seeds (including butters, pastes)	13	3.0	74.5	16	2.6	74.5	8	3.8	74.5
Potatoes (white)	14	2.8	77.3	13	2.8	77.3	12	2.8	77.3
Tomatoes, tomato/vegetable juice	15	2.8	80.1	14	2.7	80.0	11	2.9	80.2
Crackers, popcorn, pretzels, chips	16	2.5	82.6	15	2.6	82.6	14	2.4	82.6

[a] Food groups (n = 7) contributing at least 1% in descending order: Pasta; Rice, cooked grains; Fruit; Mixtures, mostly grain; Soup, broth, bouillon; Fruit drinks and ades; and Other vegetables; [b] Data are Day 1 intakes by adults aged 19+ years (n = 9490); [c] Data are Day 1 intakes by adults aged 19–50 years (n = 5429); [d] Data are Day 1 intakes by adults aged 51+ years (n = 4061).

Table S11. Food sources of vitamin B_6 among US adults (from NHANES 2003–2006).

Food group [a]	19+ years [b] Vitamin B_6 (mean = 2.0 mg)			19–50 years [c] Vitamin B_6 (mean = 2.1 mg)			51+ years [d] Vitamin B_6 (mean = 1.8 mg)		
	Ranking	% Total	Cumulative %	Ranking	% Total	Cumulative %	Ranking	% Total	Cumulative %
Ready-to-eat cereal	1	12.7	12.7	1	11.1	11.1	1	15.4	15.4
Poultry	2	9.5	22.2	2	10.2	21.3	2	8.4	23.8
Beef	3	8.6	30.8	3	9.2	30.5	3	7.4	31.2
Potatoes (white)	4	6.6	37.4	4	6.5	37.0	5	6.7	37.9
Fruit	5	5.0	42.4	7	3.8	40.8	4	7.1	45.0
Alcoholic beverages	6	4.1	46.5	5	5.1	45.9	14	2.4	47.4
Pork, ham, bacon	7	3.9	50.4	6	3.9	49.8	6	4.0	51.4
Milk	8	3.3	53.7	8	3.2	53.0	7	3.4	54.8
Other vegetables	9	3.1	56.8	9	3.1	56.1	9	3.1	57.9
Tomatoes, tomato/vegetable juice	10	3.1	59.9	10	3.1	59.2	8	3.1	61.0
Fruit juice	11	2.9	62.8	13	2.9	62.1	10	3.0	64.0
Frankfurters, sausages, luncheon meats	12	2.8	65.6	12	2.9	65.0	12	2.6	66.6
Crackers, popcorn, pretzels, chips	13	2.7	68.3	11	2.9	67.9	15	2.1	68.7
Yeast breads and rolls	14	2.5	70.8	15	2.3	70.2	11	2.9	71.6
Cake, cookies, quick bread, pastry, pie	15	2.5	73.3	14	2.7	72.9	16	2.1	73.7
Biscuits, corn bread, pancakes, tortillas	16	2.1	75.4	16	2.1	75.0	17	2.0	75.7
Fish and shellfish	17	2.1	77.5	18	1.9	76.9	13	2.4	78.1

[a] Food groups (n = 9) contributing at least 1% in descending order: Nuts, seeds (including butters, pastes); Legumes; Fruit drinks and ades; Eggs; Rice, cooked grains; Coffee, tea, other, nonalcoholic bevg.; Salad dressings, mayonnaise; Margarine and butter; and Cheese; [b] Data are Day 1 intakes by adults aged 19+ years (n = 9490); [c] Data are Day 1 intakes by adults aged 19–50 years (n = 5429); [d] Data are Day 1 intakes by adults aged 51+ years (n = 4061).

Table S12. Food sources of folate (dietary folate equivalents; DFE) among US adults (from NHANES 2003–2006).

Food group [a]	19+ years [b] Folate (mean = 407 µg DFE)			19–50 years [c] Folate (mean = 422 µg DFE)			51+ years [d] Folate (mean = 383 µg DFE)		
	**Ranking**	**% Total**	**Cumulative %**	**Ranking**	**% Total**	**Cumulative %**	**Ranking**	**% Total**	**Cumulative %**
Ready-to-eat cereal	1	18.7	18.7	1	16.7	16.7	1	22.1	22.1
Yeast breads and rolls	2	16.6	35.3	2	16.2	32.9	2	17.2	39.3
Cake, cookies, quick bread, pastry, pie	3	6.1	41.4	5	6.2	39.1	3	5.9	45.2
Pasta	4	5.7	47.1	6	5.9	45.0	4	5.3	50.5
Flour, bran, baking ingredients	5	5.6	52.7	3	6.7	51.7	6	3.6	54.1
Biscuits, corn bread, pancakes, tortillas	6	5.4	58.1	4	6.3	58.0	5	4.0	58.1
Rice, cooked grains	7	4.6	62.7	7	5.2	63.2	7	3.5	61.6
Crackers, popcorn, pretzels, chips	8	3.0	65.7	8	3.2	66.4	9	2.8	64.4
Fruit juice	9	2.6	68.3	10	2.5	68.9	10	2.7	67.1
Coffee, tea, other, nonalcoholic bevg.	10	2.5	70.8	12	2.2	71.1	8	3.1	70.2
Legumes	11	2.5	73.3	9	2.6	73.7	14	2.2	72.4
Other vegetables	12	2.3	75.6	13	2.1	75.8	11	2.7	75.1
Alcoholic beverages	13	2.0	77.6	11	2.5	78.3	23	1.0	76.1
Broccoli, spinach, greens	14	2.0	79.6	15	1.8	80.1	13	2.2	78.3

[a] Food groups (n = 10) contributing at least 1% in descending order: Fruit; Milk; Mixtures, mostly grains; Lettuce; Eggs; Nuts, seeds (including butters, pastes); Potatoes (white); Tomatoes, tomato/vegetable juices; Hot breakfast cereals; and Corn, peas, lima beans; [b] Data are Day 1 intakes by adults aged 19+ years (n = 9490); [c] Data are Day 1 intakes by adults aged 19–50 years (n = 5429); [d] Data are Day 1 intakes by adults aged 51+ years (n = 4061).

Table S13. Food sources of vitamin B_{12} among US adults (from NHANES 2003–2006).

Food group [a]	19+ years [b] Vitamin B_{12} (mean = 5.4 μg)			19–50 years [c] Vitamin B_{12} (mean = 5.6 μg)			51+ years [d] Vitamin B_{12} (mean = 5.1 μg)		
	Ranking	% Total	Cumulative %	Ranking	% Total	Cumulative %	Ranking	% Total	Cumulative %
Beef	1	19.8	19.8	1	21.4	21.4	1	17.0	17.0
Milk	2	14.7	34.5	2	14.6	36.0	2	15.0	32,0
Ready-to-eat cereal	3	12.5	47.0	3	11.2	47.2	3	14.6	46.6
Fish and shellfish	4	11.5	58.5	4	10.1	57.3	4	14.0	60.6
Cheese	5	6.7	65.2	5	7.5	64.8	5	5.3	65.9
Frankfurters, sausages, luncheon meats	6	4.7	69.9	6	4.7	69.5	7	4.6	70.5
Eggs	7	4.2	74.1	7	3.8	73.3	6	4.7	75.2
Organ meats	8	3.5	77.6	8	3.3	76.6	8	3.8	79.0
Pork, ham, bacon	9	2.7	80.3	10	2.7	79.3	9	2.6	81.6
Poultry	10	2.6	82.9	9	2.8	82.1	11	2.1	83.7

[a] Food groups (n = 6) contributing at least 1% in descending order: Cake, cookies, quick bread, pastry, pie; Milk desserts; Mixtures, mostly grains; Milk drinks; Margarine and butter; and Fruit drinks and ades; [b] Data are Day 1 intakes by adults aged 19+ years (n = 9490); [c] Data are Day 1 intakes by adults aged 19–50 years (n = 5429); [d] Data are Day 1 intakes by adults aged 51+ years (n = 4061).

Table S14. Food sources of phosphorus among US adults (from NHANES 2003–2006).

**Food group** [a]	**19+ years** [b]			**19–50 years** [c]			**51+ years** [d]		
	**Phosphorus (mean = 1350 mg)**			**Phosphorus (mean = 1436 mg)**			**Phosphorus (mean = 1215 mg)**		
	**Ranking**	**% Total**	**Cumulative %**	**Ranking**	**% Total**	**Cumulative %**	**Ranking**	**% Total**	**Cumulative %**
Milk	1	12.3	12.3	2	11.9	11.9	1	13.1	13.1
Cheese	2	11.3	23.6	1	12.5	24.4	2	9.2	22.3
Beef	3	6.7	30.3	4	7.0	31.4	3	6.2	28.5
Poultry	4	6.6	36.9	3	7.1	38.5	4	5.8	34.3
Biscuits, corn bread, pancakes, tortillas	5	4.6	41.5	5	4.9	43.4	6	4.1	38.4
Yeast breads and rolls	6	4.5	46.0	6	4.0	47.4	5	5.4	43.8
Pork, ham, bacon	7	3.8	49.8	7	3.7	51.1	7	3.9	47.4
Cake, cookies, quick bread, pastry, pie	8	3.6	53.4	8	3.5	54.6	8	3.9	51.6
Fish and shellfish	9	3.2	56.6	9	2.9	57.5	9	3.9	55.5
Eggs	10	2.9	59.5	13	2.6	60.1	10	3.4	58.9
Frankfurters, sausages, luncheon meats	11	2.8	62.3	10	2.9	63.0	13	2.6	61.5
Nuts, seeds (including butters, pastes)	12	2.6	64.9	16	2.3	65.3	11	3.1	64.6
Crackers, popcorn, pretzels, chips	13	2.5	67.4	12	2.7	68.0	15	2.3	66.9
Soft drinks, soda (including diet)	14	2.3	69.7	11	2.7	70.7	18	1.7	68.6
Ready-to-eat cereal	15	2.3	72.0	18	1.8	72.5	12	3.1	71.7
Potatoes (white)	16	2.3	74.3	15	2.4	74.9	16	2.1	73.8
Milk desserts	17	2.0	76.3	19	1.7	76.6	14	2.6	76.4
Alcoholic beverages	18	2.0	78.3	14	2.4	79.0	23	1.2	77.6

[a] Food groups (*n* = 10) contributing at least 1% in descending order: Legumes; Flour, bran, baking ingredients; Pasta; Mixtures, mostly grain; Tomatoes, tomato/vegetable juice; Other vegetables; Candy, sugars and sugary foods; Fruit; Milk drinks; and Hot breakfast cereal; [b] Data are Day 1 intakes by adults aged 19+ years (*n* = 9490); [c] Data are Day 1 intakes by adults aged 19–50 years (*n* = 5429); [d] Data are Day 1 intakes by adults aged 51+ years (*n* = 4061).

Table S15. Food sources of magnesium among US adults (from NHANES 2003–2006).

**Food group** [a]	**19+ years** [b]			**19–50 years** [c]			**51+ years** [d]		
	**Magnesium (mean = 292 mg)**			**Magnesium (mean = 303 mg)**			**Magnesium (mean = 275 mg)**		
	**Ranking**	**% Total**	**Cumulative %**	**Ranking**	**% Total**	**Cumulative %**	**Ranking**	**% Total**	**Cumulative %**
Coffee, tea, other, nonalcoholic bevg.	1	9.2	9.2	1	8.4	8.4	1	10.4	10.4
Milk	2	6.5	15.7	2	6.4	14.8	3	6.6	17.0
Yeast breads and rolls	3	6.0	21.7	3	5.4	20.2	2	6.9	23.9
Nuts, seeds (including butters, pastes)	4	5.2	26.9	6	4.6	24.8	4	6.3	30.2
Crackers, popcorn, pretzels, chips	5	4.7	31.6	4	5.3	30.1	7	3.8	34.0
Alcoholic beverages	6	4.0	35.6	5	4.9	35.0	16	2.3	36.3
Cake, cookies, quick bread, pastry, pie	7	3.8	39.4	8	4.0	39.0	9	3.6	39.9
Poultry	8	3.8	43.2	7	4.2	43.2	10	3.2	43.1
Potatoes (white)	9	3.7	46.9	9	3.7	36.9	8	3.6	46.7
Fruit	10	3.5	50.4	17	2.7	49.6	5	4.9	51.6
Ready-to-eat cereal	11	3.4	53.8	15	2.8	52.4	6	4.5	56.1
Beef	12	3.3	57.1	11	3.6	56.0	11	2.9	59.0
Biscuits, corn bread, pancakes, tortillas	13	3.1	60.2	10	3.6	59.6	20	2.1	61.1
Legumes	14	3.0	63.2	13	3.2	62.8	12	2.8	63.9
Cheese	15	2.9	66.1	12	3.2	66.0	18	2.2	66.1
Tomatoes, tomato/vegetable juice	16	2.9	69.0	14	2.9	68.9	13	2.7	68.8
Fruit juice	17	2.7	71.7	16	2.8	71.7	14	2.7	71.5
Fish and shellfish	18	2.3	74.0	18	2.2	73.9	15	2.5	74.0
Other vegetables	19	2.1	76.1	20	2.1	76.0	17	2.2	76.2

[a] Food groups (*n* = 12) contributing at least 1% in descending order: Candy, sugars and sugary foods; Pasta; Pork, ham, bacon; Milk desserts; Rice, cooked grains; Broccoli, spinach, greens; Hot breakfast cereal; Flour, bran, baking ingredients; Frankfurters, sausages, luncheon meats; Corn, peas, lima beans; Condiments and sauces; and Meal replacements/supplements; [b] Data are Day 1 intakes by adults aged 19+ years (*n* = 9490); [c] Data are Day 1 intakes by adults aged 19–50 years (*n* = 5429); [d] Data are Day 1 intakes by adults aged 51+ years (*n* = 4061).

Table S16. Food sources of iron among US adults (from NHANES 2003–2006).

Food group [a]	19+ years [b] Iron (mean = 15.2 mg)			19–50 years [c] Iron (mean = 15.2 mg)			51+ years [d] Iron (mean = 15.2 mg)		
	Ranking	% Total	Cumulative %	Ranking	% Total	Cumulative %	Ranking	% Total	Cumulative %
Ready-to-eat cereal	1	15.1	15.1	1	13.0	13.0	1	18.8	18.8
Yeast breads and rolls	2	12.5	27.6	2	12.0	25.0	2	13.4	32.2
Beef	3	6.9	34.5	3	7.3	32.3	3	6.1	38.3
Cakes, cookies, quick bread, pastry, pie	4	6.1	40.6	4	6.1	38.4	4	6.1	44.4
Biscuits, corn bread, pancakes, tortillas	5	4.7	45.3	5	5.3	43.7	5	3.7	48.1
Flour, bran, baking ingredients	6	4.4	49.7	6	5.2	48.9	8	2.9	51.0
Poultry	7	3.3	53.0	7	3.6	52.5	9	2.7	53.7
Crackers, popcorn, pretzels, chips	8	3.2	56.2	8	3.4	55.9	7	2.9	56.6
Tomatoes, tomato/vegetable juice	9	2.9	59.1	10	3.0	58.9	12	2.6	59.2
Legumes	10	2.7	61.8	11	2.8	61.7	10	2.7	61.9
Soft drinks, soda (includes diet)	11	2.6	64.4	9	3.2	64.9	19	1.7	63.6
Pasta	12	2.3	66.7	12	2.4	67.3	13	2.1	65.7
Eggs	13	2.3	69.0	15	2.1	69.4	11	2.6	68.3
Rice, cooked grains	14	2.1	71.1	13	2.4	71.8	20	1.6	69.9

[a] Food groups (n = 13) contributing at least 1% in descending order: Frankfurters, sausages, luncheon meats; Potatoes (white); Hot breakfast cereal; Fruit juice; Fish and shellfish; Nuts, seeds (including butters, pastes); Mixtures, mostly grain; Meal replacements/supplements; Pork, ham, bacon; Fruit; Other vegetables; Soup, broth, bouillon; and Candy, sugars and sugary foods; [b] Data are Day 1 intakes by adults aged 19+ years (n = 9490); [c] Data are Day 1 intakes by adults aged 19–50 years (n = 5429); [d] Data are Day 1 intakes by adults aged 51+ years (n = 4061).

Table S17. Food sources of zinc among US adults (from NHANES 2003–2006).

**Food group** [a]	**19+ years** [b]			**19–50 years** [c]			**51+ years** [d]		
	**Zinc (mean = 12.5 mg)**			**Zinc (mean = 13.3 mg)**			**Zinc (mean = 11.2 mg)**		
	**Ranking**	**% Total**	**Cumulative %**	**Ranking**	**% Total**	**Cumulative %**	**Ranking**	**% Total**	**Cumulative %**
Beef	1	21.1	21.1	1	22.0	22.0	1	19.4	19.4
Ready-to-eat cereal	2	7.8	28.9	3	6.7	28.7	2	9.8	29.2
Cheese	3	7.4	36.3	2	8.1	36.8	4	5.9	35.1
Poultry	4	6.2	42.5	4	6.5	43.3	5	5.5	40.6
Milk	5	5.9	48.4	5	5.7	49.0	3	6.3	46.9
Yeast breads and rolls	6	4.2	52.6	9	3.9	52.9	6	5.0	51.9
Frankfurters, sausages, luncheon meats	7	4.0	56.6	6	4.1	57.0	9	3.9	55.8
Pork, ham, bacon	8	4.0	60.6	7	3.9	60.9	7	4.2	60.0
Fish and shellfish	9	4.0	64.6	8	3.9	64.8	8	4.1	64.1
Legumes	10	2.8	67.4	11	2.7	67.5	10	3.0	67.1
Crackers, popcorn, pretzels, chips	11	2.7	70.1	10	2.9	70.4	13	2.3	69.4
Cake, cookies, quick bread, pastry, pie	12	2.5	72.6	12	2.5	72.9	12	2.4	71.8
Nuts, seeds (including butters, pastes)	13	2.4	75.0	13	2.0	74.9	11	3.0	74.8

[a] Food groups (n = 13) contributing at least 1% in descending order: Eggs; Biscuits, corn bread, pancakes, tortillas; Mixtures, mostly grain; Potatoes (white); Milk desserts; Candy, sugars and sugary foods; Pasta; Coffee, tea, other, nonalcoholic beverages; Tomatoes, tomato/vegetable juice; Rice, cooked grains; Flour, bran, baking ingredients; Meal replacements/supplements; and Other vegetables; [b] Data are Day 1 intakes by adults aged 19+ years (n = 9490); [c] Data are Day 1 intakes by adults aged 19–50 years (n = 5429); [d] Data are Day 1 intakes by adults aged 51+ years (n = 4061).
